# Supplementary material for: Lipidomics of Caco-2 Cells Under Simulated Microgravity Conditions
Source: Int J Mol Sci. 2024 Nov 25;25(23):12638. doi: 10.3390/ijms252312638 (PMC11641246; doi:10.3390/ijms252312638)
Supplement: Supplementary file 1 [file ijms-25-12638-s001.zip › ijms-3221507-supplementary.pdf]

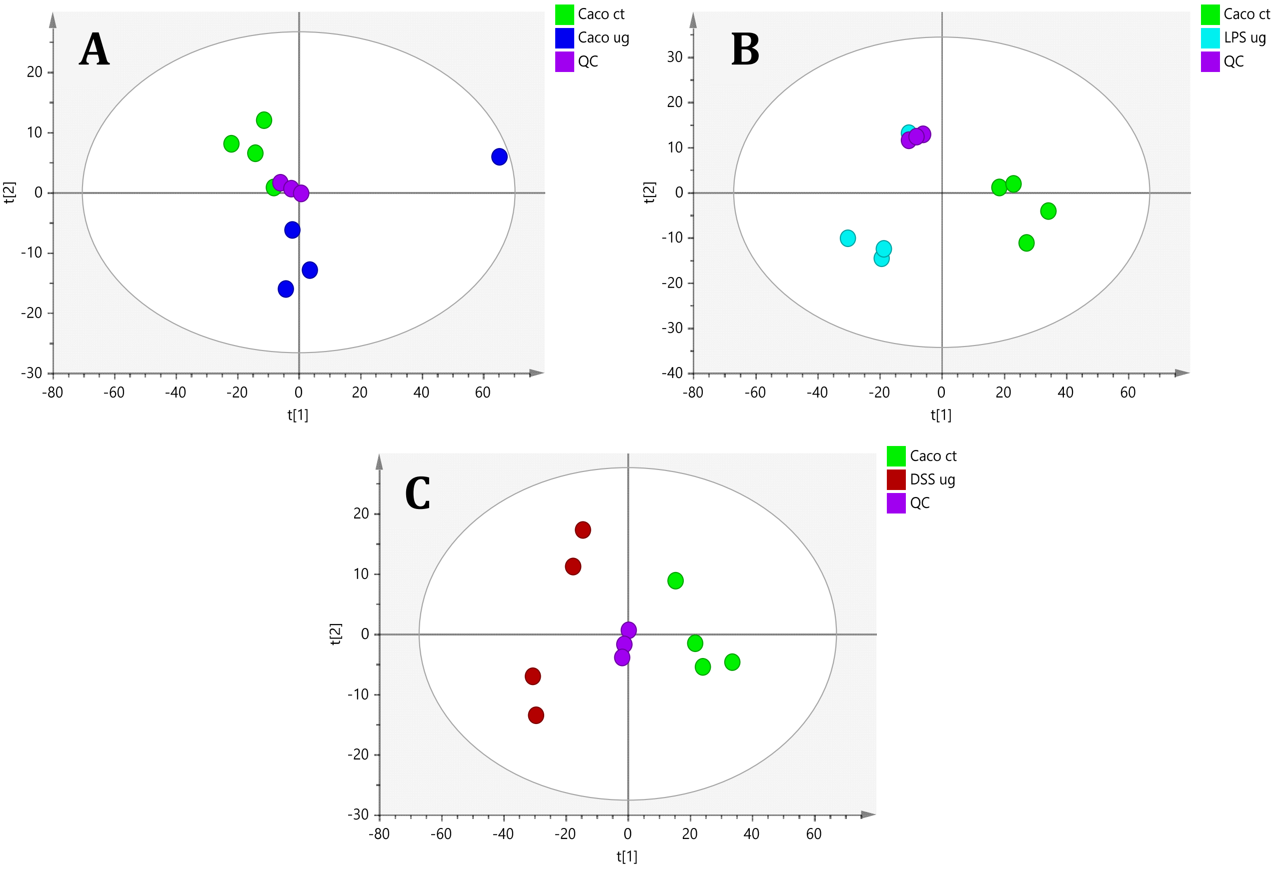

Figure S1: PCA score plots of Caco-2 cells subjected to simulated microgravity conditions (A), Caco-2 cells co-cultured with DSS (B) and LPS (C) under simulated microgravity conditions compared to controls. Validation parameters for the statistical model are indicated with R^2X and Q^2 values.

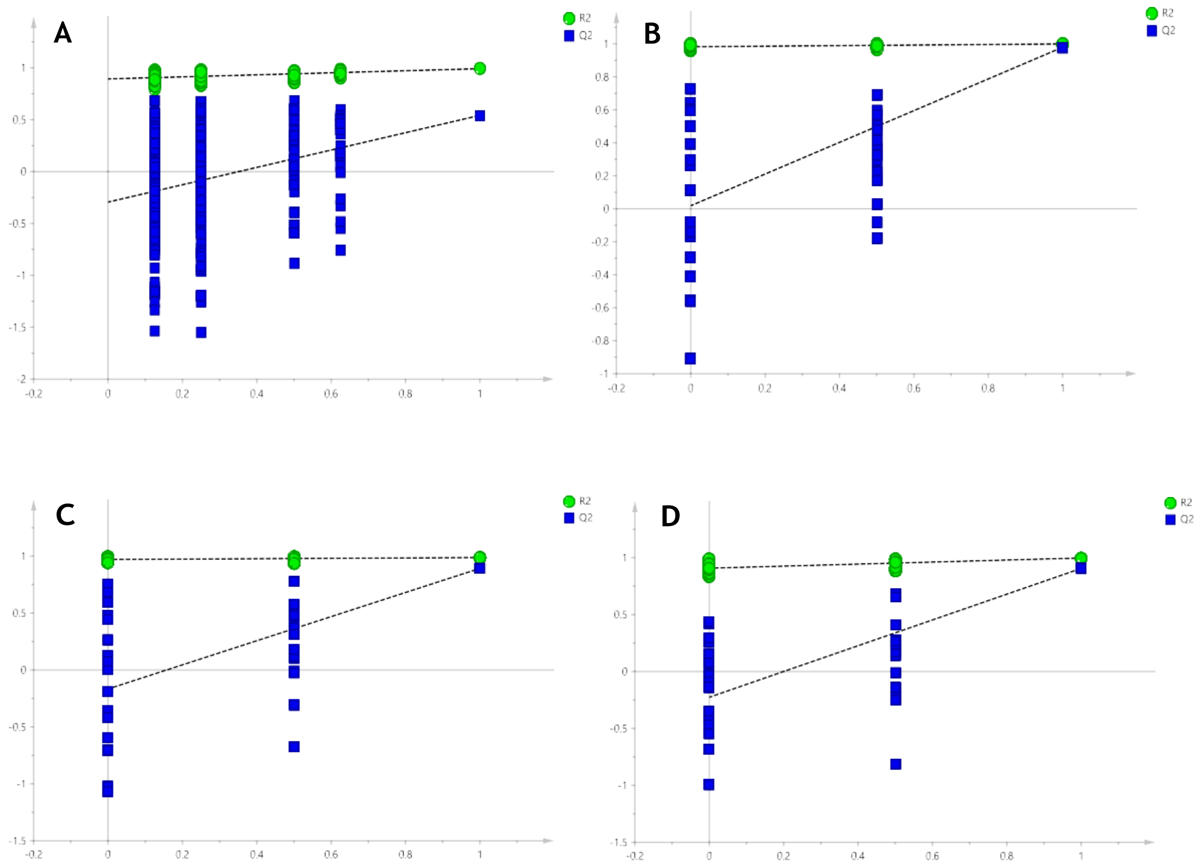

Figure S2. Statistical significance of the permutation tests conducted on the Caco-2 cell experiment treated with DSS (A), DSS in simulated microgravity (B), LPS (C) and LPS in simulated microgravity (D).

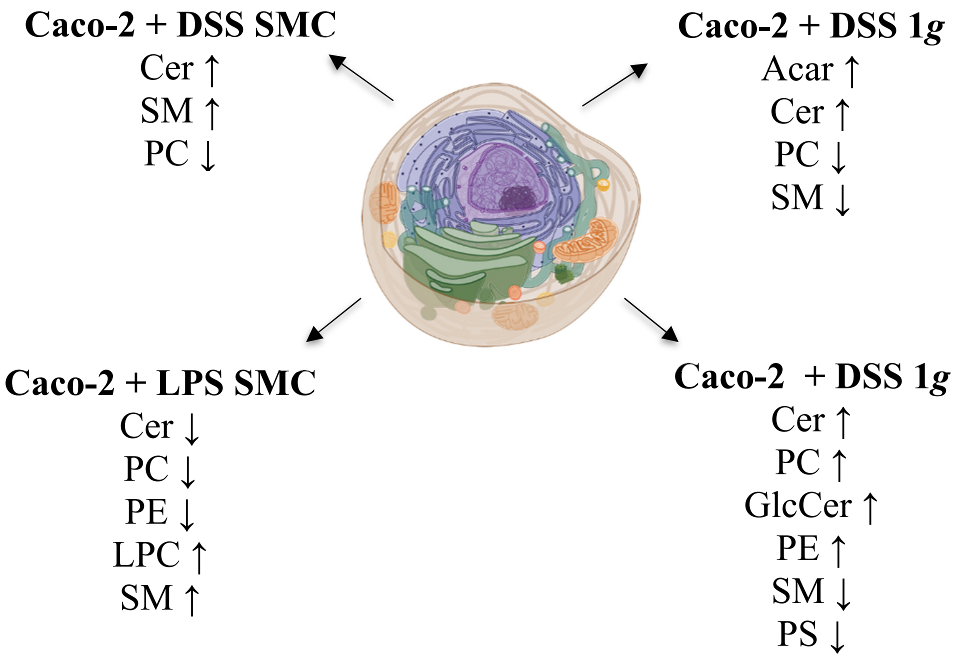

Figure S3: The cartoon illustrates the discriminant outcomes related to changes in lipid regulation observed during experiments conducted under simulated microgravity (SMC) and terrestrial gravity (1g) conditions.

Table S1. Primer sequences for genes target used in amplification: Forward and reverse primers listed for each target to ensure specific and efficient gene amplification.

**Gene Target**	**Forward primer (5–3′)**	**Reverse primer (5–3′)**
**SPTLC1**	GCAGTGTTGAAGGAAAAGTGCGG	CAGTGCTCTCTTCCAGTTGTAGG
**SPTLC2**	CCAGACTGTCAGGAGCAACCAT	TTCGTGTCCGAGGCTGACCATA
**KDSR**	CGGTTTCACAGCCTACTCTGCA	GTTTTCTTCGGCAAAGCCAGGTG
**CERS2**	GCCTTGCTCTTCCTCATCGTTC	TGCTTGCCACTGGTCAGGTAGA
**CERS6**	GACGCAATCAGGAGAAGCCAAG	GGTAGTTGTACCAGCAATGCCTC
**DEGS1**	CCAACATTCCTGGAAAAAGTCTTC	GCCTCTTCATTCTTGAGTAGGGA

Table S2. Differential gene expression related to sphingolipid metabolism in Caco-2 cells under simulated microgravity condition (SMC) and terrestrial gravity conditions following DSS and LPS treatments. Gene expression changes are presented as statistically significant differences compared to untreated controls, with significance determined by the T-test. Symbols represent levels of significance: *p < 0.05, **p < 0.01, and ***p < 0.001. "ns" indicates nonsignificant changes.

	**SMC**	
	DSS vs Ctrl	LPS vs Ctrl
SPTL C1	**	ns
SPTL C2	ns	*
KDSR	*	*
Cer S2	*	*
Cer S6	**	*
DEG S1	*	***
	**Terrestrial gravity**	
	DSS vs Ctrl	LPS vs Ctrl
SPTL C1	**	*
SPTL C2	**	*
KDSR	**	ns
Cer S2	**	*
Cer S6	**	**
DEG S1	***	**

Table S3. Lipids and their respective hydrophobic chain composition, precursor ion (*m/z*), product ion (*m/z*) and collision energy (V).

Lipid class	Attribution	Precursor ion *(m/z)*	Product ions *(m/z)*	CE (V)
**Cer**	18:0;O2/12:0	484.50	466.50	20
	18:0;O2/16:0	540.50	522.50	20
	18:0;O2/18:0	568.50	550.50	20
	18:0;O2/24:0	652.50	634.50	20
	18:1;O2/14:0	510.48	264.30	20
	18:1;O2/15:0	524.50	264.30	20
	18:1;O2/16:0	538.50	264.30	20
	18:1;O2/16:1	536.50	264.30	20
	18:1;O2/17:0	574.51	264.30	20
	18:1;O2/18:0	548.00	264.20	20
	18:1;O2/18:1	563.50	264.30	20
	18:1;O2/ 20:0	594.60	264.30	20
	18:1;O2/ 22:0	604.30	264.20	20
	18:1;O2/22:1	620.60	264.30	20
	18:1;O2/ 23:0	636.60	264.30	20
	18:1;O2/23:1	634.60	264.30	20
	18:1;O2/24:0	652.50	632.50	20
	18:1;O2/24:1	648.60	264.30	20
	18:1;O2/26:0	678.67	264.27	20
	18:2;O2/16:0	536.50	262.25	20
	18:2;O2/22:0	620.59	262.30	20
	18:2;O2/24:1	646.60	262.30	20
	18:2;O2/26:2	672.60	262.30	20
**SM**	18:1;O2/14:0	675.50	184.10 / 264.27	30
	18:1;O2/15:0	689.60	184.10 / 264.27	30
	18:1;O2/15:1	687.50	184.10 / 264.27	30
	18:1;O2/16:0	703.20	184.10 / 264.27	20
	18:1;O2/16:1	701.60	184.10 / 264.27	30
	18:1;O2/17:0	717.60	184.10 / 264.27	30
	18:1;O2/18:0	731.50	184.10 / 264.27	20
	18:1;O2/19:0	745.60	184.10 / 264.27	30
	18:1;O2/20:0	759.60	184.10 / 264.27	30
	18:1;O2/20:1	757.60	184.10 / 264.27	30
	18:1;O2/21:0	773.70	184.10 / 264.27	30
	18:1;O2/21:1	771.60	184.10 / 264.27	30
	18:1;O2/22:0	787.70	184.10 / 264.27	30
	18:1;O2/22:1	785.70	184.10 / 264.27	30
	18:1;O2/22:2	783.60	184.10 / 264.27	30
	18:1;O2/23:0	801.70	184.10 / 264.27	30

	18:1;O2/23:1	799.70	184.10 / 264.27	30
	18:1;O2/23:2	797.70	184.10 / 264.27	30
	18:1;O2/24:0	815.10	184.10 / 264.27	25
	18:1;O2/24:1	813.20	184.10 / 264.27	22
	18:1;O2/24:2	811.70	184.10 / 264.27	30
	18:1;O2/24:3	809.70	184.10 / 264.27	30
	18:1;O2/25:0	829.70	184.10 / 264.27	30
	18:1;O2/25:1	827.70	184.10 / 264.27	30
	18:1;O2/26:0	843.70	184.10 / 264.27	30
	18:2;O2/16:0	701.56	262.24	30
	18:2;O2/18:0	729.59	184.07 / 262.24	30
	18:2;O2/18:0	729.59	262.24	30
	18:2;O2/20:0	757.62	262.24	30
	18:2;O2/21:0	771.63	184.07 / 262.24	30
	18:2;O2/22:0	785.65	184.07 / 262.24	30
	18:2;O2/23:0	799.66	184.07 / 262.24	30
	18:2;O2/24:0	813.67	184.07 / 262.24	30
	18:2;O2/24:2	809.65	262.24	30
	18:2;O2/24:3	807.63	184.07 / 262.24	30
	18:2;O2/25:0	827.69	184.07 / 262.24	30
**GlcCer**	18:1;O2/12:0	626.2	264.20	25
	18:1;O2/16:0	682.2	264.20	25
	18:1;O2/18:0	710.1	264.20	25
	18;1;O2/18:1	708.1	264.20	25
	18;1;O2/24:1	792.1	264.20	25
**SPH**	17:0	288.1	270.10	20
	17:1	286.1	268.30	20
	18:0	300.20	284.20	15
	18:1	300.2	282.20	12

Table S4. Discriminating lipids annotated when Caco-2 cells were treated with DSS under terrestrial gravity conditions (1 *g*)

Attribution	Adduct	*Theoretical (m/z)*	*Experimental (m/z)*	*Δ ppm*	RT (*min*)	VIP	Regulation at 1 *g*
ACar 14:0	(M+H)+	372.3108	372.3104	-1.07	0.85	1.10	+
ACar 14:1	(M+H)+	370.2952	370.2950	-1.35	0.79	1.12	+
ACar 16:0	(M+H)+	400.3421	400.3417	-0.99	1.00	1.10	+
ACar 16:1	(M+H)+	398.3265	398.3260	-1.25	0.88	1.15	+
ACar 18:1	(M+H)+	426.3578	426.3573	-1.17	1.03	1.02	+
Cer 18:1;O2/16:0	(M+H)+	538.5194	538.5191	-0.52	4.24	1.01	+
Cer 18:0;O2/22:0	(M+H)+	624.6289	624.6290	172	5.51	1.02	+
Cer 18:1;O2/24:0	(M+H)+	650.6446	650.6441	-0.43	8.12	1.25	+
Cer 18:2;O2/24:0	(M+H)+	648.6289	648.6285	-0.46	7.84	1.01	+
PC 14:0/16:1	(M+H)+	704.5225	704.5220	0.14	3.01	1.30	-
PC 16:0/16:1	(M+H)+	732.5538	732.5536	1.91	3.20	1.80	-
PC 16:1/16:1	(M+H)+	730.5381	730.5383	1.36	2.65	1.67	-
PC 17:1/18:1	(M+H)+	772.5851	772.5850	1.29	5.29	1.44	-
PC 18:0/18:1	(M+H)+	788.6164	788.6163	1.77	5.65	1.35	-
PC 18:2/18:3	(M+H)+	780.5538	780.5532	0.64	3.25	1.52	-
PC 18:3/18:3	(M+H)+	778.5381	778.5386	-0.25	4.20	1.31	-
PC 18:1/20:0	(M+H)+	816.6477	816.6471	0.01	4.57	1.42	-
PC 18:1/20:1	(M+H)+	814.6320	814.6323	0.85	5.68	1.29	-
SM 18:1;O2/16:0	(M+H)+	703.5748	703.5744	1.70	3.43	1.73	-
SM 18:1;O2/16:1	(M+H)+	701.5592	701.5596	-0.14	2.83	1.27	-
SM 18:0;O2/22:0	(M+H)+	789.6844	789.6842	0.12	7.16	1.61	-
SM 18:1;O2/22:1	(M+H)+	785.6531	785.6530	0.01	5.46	1.39	-
SM 18:1;O2/23:0	(M+H)+	801.6844	801.6845	-5.29	7.35	1.57	-
SM 18:2;O2/23:0	(M+H)+	799.6687	799.6686	-0.25	6.30	1.38	-
SM 18:1;O2/24:0	(M+H)+	815.7000	815.7003	1.10	7.82	1.76	-
SM 18:1;O2/24:1	(M+H)+	813.6844	813.6840	-0.49	6.46	1.16	-

Table S5. Discriminating lipids annotated in the lipidomic analysis of Caco-2 cells treated with LPS under terrestrial conditions.

Attribution	Adduct	*Theoretical (m/z)*	*Experimental (m/z)*	*Δ ppm*	RT (*min*)	VIP	Regulation at 1 *g*
Cer 18:0;O2/16:0	(M-H)-	538.5205	538.5201	0.55	4.59	1.10	+
Cer 18:1;O2/16:0	(M+H)+	538.5194	538.5198	-0.52	4.24	1.01	+
Cer 18:1;O2/24:0	(M+H)+	650.6446	650.6441	-0.94	8.14	1.03	+
Cer 18:2; O2/24:0	(M+H)+	648.6289	648.6287	-0.77	7.84	1.02	+
GlcCer 18:1;O2/16:0	(M+H)+	700.5722	700.5725	0.42	3.90	1.18	+
PC 15:1/16:0	(M+H)+	718.5381	718.5384	0.55	4.73	1.28	+
PC 15:1/16:1	(M+H)+	716.5225	716.5222	0.69	3.94	1.50	+
PC 18:1/14:0	(M+H)+	732.5538	732.5534	-0.27	4.82	1.13	+
PC 15:1/18:0	(M+H)+	746.5694	746.5693	0.40	4.24	1.15	+
PC 18:0/16:0	(M+H)+	762.6007	762.6001	-0.39	6.01	1.13	+
PC 15:0/20:5	(M+H)+	766.5381	766.5384	0.65	3.97	1.36	+
PC 22:1/14:0	(M+H)+	788.6164	788.6162	0.63	5.76	1.62	+
PC 18:1/18:1	(M+H)+	786.6007	786.6001	-0.38	4.77	1.34	+
PC 18:1/20:1	(M+H)+	814.6320	814.6329	0.73	5.80	1.33	+
PE 18:1/18:0	(M+H)+	746.5694	746.5692	-0.13	4.24	1.42	+
PE 18:1/20:0	(M+H)+	774.6007	774.6003	0.51	5.19	1.34	+
PE 18:1/20:1	(M+H)+	772.5851	772.5850	-0.25	4.33	1.10	+
PS 16:0/17:0	(M+H)+	750.5280	750.5285	0.79	5.99	1.26	-
SM 18:0;O2/16:0	(M+H)+	705.5905	705.5900	-0.56	3.60	1.21	-
SM 18:1;O2/16:0	(M+H)+	703.5748	703.5743	0.42	3.43	1.53	-
SM 16:1;O2/24:0	$(M+H)^+$	815.7000	815.7002	0.36	7.82	1.18	-
SM 18:2;O2/24:0	$(M+H)^+$	813.6844	813.6840	-0.73	6.46	1.10	-
